# Intracortical Brain-Machine Interfaces Advance Sensorimotor Neuroscience

**DOI:** 10.3389/fnins.2016.00291

**Published:** 2016-06-28

**Authors:** Karen E. Schroeder, Cynthia A. Chestek

**Affiliations:** ^1^Department of Biomedical Engineering, University of MichiganAnn Arbor, MI, USA; ^2^Neuroscience Graduate Program, University of Michigan Medical SchoolAnn Arbor, MI, USA; ^3^Center for Consciousness Science, University of Michigan Medical SchoolAnn Arbor, MI, USA; ^4^Department of Electrical Engineering and Computer Science, University of MichiganAnn Arbor, MI, USA; ^5^Robotics Graduate Program, University of MichiganAnn Arbor, MI, USA

**Keywords:** brain-machine interface, neuroprosthetics, motor cortex, motor learning, reaching

## Abstract

Brain-machine interfaces (BMIs) decode brain activity to control external devices. Over the past two decades, the BMI community has grown tremendously and reached some impressive milestones, including the first human clinical trials using chronically implanted intracortical electrodes. It has also contributed experimental paradigms and important findings to basic neuroscience. In this review, we discuss neuroscience achievements stemming from BMI research, specifically that based upon upper limb prosthetic control with intracortical microelectrodes. We will focus on three main areas: first, we discuss progress in neural coding of reaches in motor cortex, describing recent results linking high dimensional representations of cortical activity to muscle activation. Next, we describe recent findings on learning and plasticity in motor cortex on various time scales. Finally, we discuss how bidirectional BMIs have led to better understanding of somatosensation in and related to motor cortex.

## Introduction

The particular demands of BMI experiments have produced great advances in our understanding of neural coding in sensorimotor areas. Many simultaneously recorded cells are necessary for prosthetic control, so large data sets from motor cortex and premotor areas have been produced, either with many microwires or with microelectrode arrays. Non-human primates have traditionally been used, as they share many similarities in upper limb and motor cortical anatomy with humans, have a large enough cortex in which to squeeze many channels of recordings, and can be trained on complex tasks using positive reinforcement. These data sets comfortably occupy the experimental space between humans and rodents, providing thousands of trials worth of high dimensional, low noise recordings from expertly trained animals. Many BMI labs take advantage of the large amounts of time and energy that go into training these animals by using them to answer basic science questions, as well as to improve prosthetic control. Once recorded, the data remain valuable for offline analysis, as well as validation of computational models of cortical function.

Substantial advances have been made in human cortical BMI over the past decade by multiple groups working with subjects with tetraplegia (Hochberg et al., 2006, 2012; Simeral et al., 2011; Collinger et al., 2013; Gilja et al., 2015; Jarosiewicz et al., 2015; Wodlinger et al., 2015). Human subjects in these studies have also been able to provide multiple years' worth of valuable data. While publications on this data have thus far been focused on engineering results, it seems inevitable that we will soon be seeing more analysis of human single units in the literature (for a perspective on applications of single units in neurology, see Cash and Hochberg, 2015).

In 2009, Hatsopoulos and Donoghue reviewed insights that neural interface research had contributed to neuroscience, focusing in part on population coding of movement parameters, the distributed nature of motor encoding over many frontal and parietal regions, and motor learning (Hatsopoulos and Donoghue, 2009). But progress is being made very rapidly, so we will expand upon these themes, incorporating the significant advances that have been made in the past 7 years. Other recent reviews (Wander and Rao, 2014; Moxon and Foffani, 2015; Oweiss and Badreldin, 2015; Golub et al., 2016) have done an excellent job of providing detailed histories of BMIs and the various ways in which BMI experiments can push basic neuroscience forward. To complement these efforts, we instead will detail more specific neuroscientific achievements stemming directly from BMIs and the methodologies they produced, focusing on primary motor cortex (M1) and somatosensory cortex (S1).

## Scientific contributions

### Movement coding in motor cortex

It is commonly noted that BMI experiments have provided the perfect sandbox for the testing of M1 coding schemes (Hatsopoulos and Donoghue, 2009; Georgopoulos and Carpenter, 2015). Instead of analyzing individual cells' responses to movements offline, larger populations can be used to reconstruct movement parameters in real time. Shortcomings in our understanding of whole movement encoding immediately become clear under these conditions, demanding ever more comprehensive models. The lineage of this work began with operant conditioning of single M1 units (Fetz, 1969; Fetz and Finocchio, 1971), in which monkeys could modulate the activity of individual cells to obtain juice rewards. More recently, this approach has been used successfully for cursor control (Moritz and Fetz, 2011; Milovanovic et al., 2015), but many-degree-of-freedom prosthetic control has thus far required a larger population of cells. Humphrey et al. (1970) recorded from a handful of neurons simultaneously, and were able to predict the force applied by a monkey in a wrist flexing task using a weighted sum of firing rates. Soon came a description of population coding of arm kinematics (Georgopoulos et al., 1986, 1988) – that an accurate estimate of reach direction could be drawn from a consensus of multiple individually tuned neurons. This was followed by a wave of closed-loop reaching experiments (Serruya et al., 2002; Taylor et al., 2002; Carmena et al., 2003) using M1 recordings, but there were still many open questions about the planning and generation of even a simple reach. With the basic experimental paradigm set, researchers began to explore these higher dimensional data sets. “Higher order sensory and motor representations appear to emerge from the firing of neuronal assemblies, but it has yet to be determined whether spatial and temporal interactions contribute to these representations,” stated one paper (Maynard et al., 1999), after showing that additional directional information could be extracted from second order interactions (covariance) between M1 neurons. This would turn out to be the case, and soon the importance of ensemble activity over individual cell firing rates would be explored.

**Figure 1:**
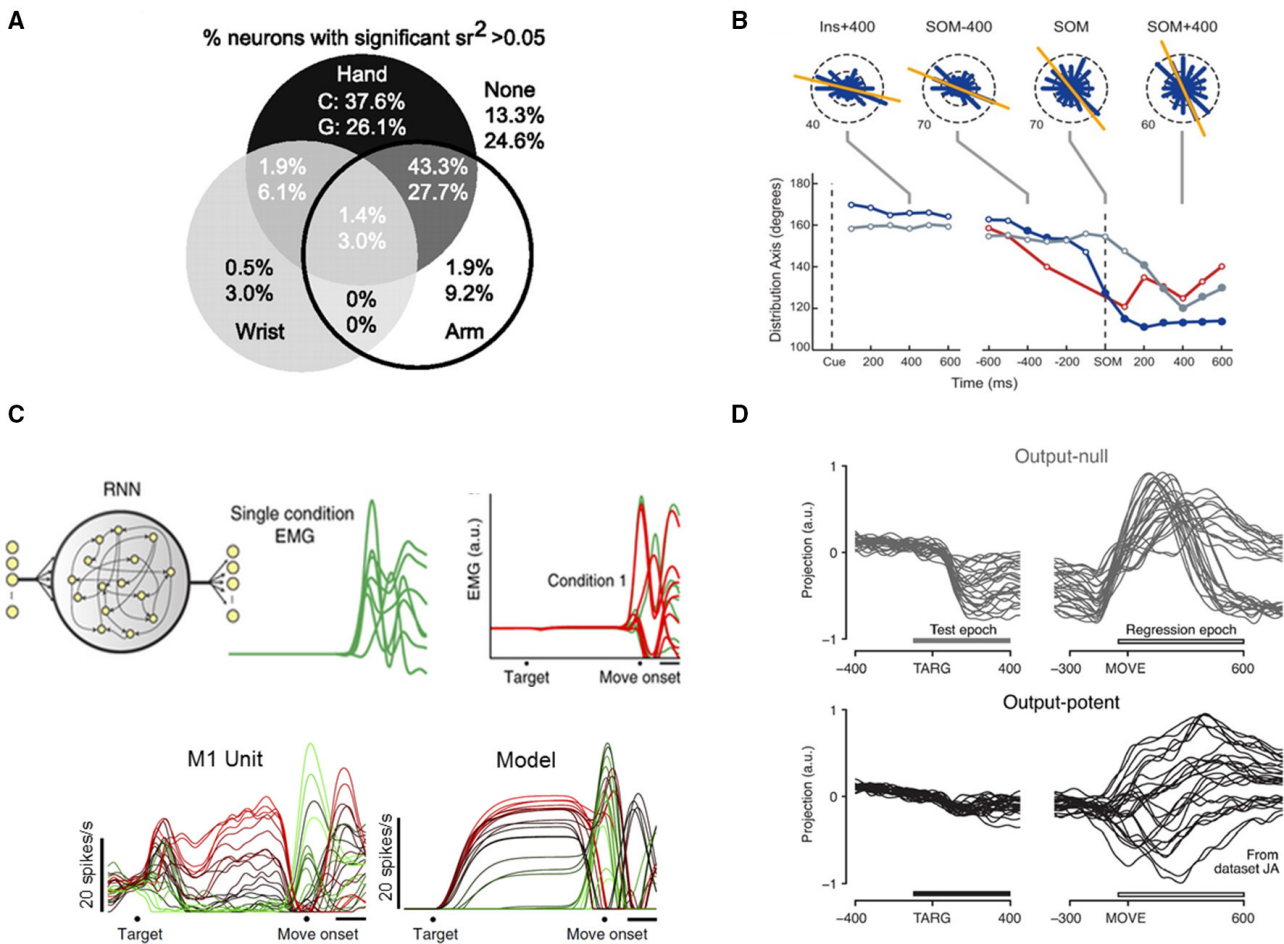
**(A)** Many M1 neurons exhibit significant semipartial correlations between firing rate and kinematics of multiple arm regions. Percentages shown separately for two monkeys, referred to as “C” and “G.” Reprinted with permission of The Society for Neuroscience, from Vargas-Irwin et al. (2010); permission conveyed through Copyright Clearance Center, Inc. **(B)**, Preferred directions of neurons change over the course of an instructed-delay reach. Top: circular frequency histograms of the preferred directions of M1 cells. Bottom: summary of the preferred direction distribution axis orientation in each area over the course of a trial for M1 (blue), PMd (gray), and PMv (red). SOM: move onset. Reprinted with permission of The American Physiological Society, from Suminski et al. (2015). **(C)**, A recurrent neural network optimized to generate EMG finds solutions similar to native M1 neurons. Top left: Network inputs consisted of a condition-independent hold cue (purple) and a six-dimensional condition-specific input (black), which specified the condition (reach) for which the network should generate EMG. Top right: An example condition showing the multiple muscle target EMG (green, one trace per muscle) and the corresponding trained outputs of the regularized model (red). Bottom: Example peri-stimulus time histograms from one M1 neuron and one model neuron; each trace represents one of 27 conditions (reaches). Adapted with permission of Macmillan Publishers Ltd., from Sussillo et al. (2015), copyright 2015. **(D)**, Tuned preparatory activity in an output-null dimension. Trial-averaged neural activity in one output-null and one output-potent dimension are shown, one trace per condition (reach). This pair of example dimensions has a tuning ratio of 9.2. Bars indicate “test epoch” (−100 to +400 ms from target onset), where the tuning ratio was computed, and “regression epoch” (−50 to +600 ms from movement onset), where dimensions were identified. Reprinted with permission of Macmillan Publishers Ltd., from Kaufman et al. (2014), copyright 2014.

Another important contribution of the field with roots in this time period is the lack of precise M1 somatotopy. Major bodily areas are segregated, but smaller regions intermingle (Sanes and Donoghue, 2000; Sanes and Schieber, 2001; Schieber, 2001), with substantial overlap of, for example, muscles and joints of the hand and fingers. It is now expected that an array placed in hand representation of macaque M1 will produce a disorderly mixture of digit preferences. This is important to know for surgical implant logistics, but also points to the multifaceted response properties of M1 output cells. We know that M1 neurons exhibit both convergence and divergence: individual neurons diverge to innervate multiple muscles, and many M1 cells converge to innervate any given muscle. In keeping with this, several more recent studies have shown that both reaching and grasping can be decoded from the same (relatively) small population of cells on a single array (Carmena et al., 2003; Velliste et al., 2008; Vargas-Irwin et al., 2010). The latter study demonstrated that cells recorded from one 4 × 4 mm array could reconstruct 25 joint angles encompassing the hand, wrist and arm, and that individual cells often represented both proximal and distal joints (Figure 1A). In this study, the arrays were targeted at the upper-limb region of M1, but many cells from both monkeys displayed variances in firing rates that were significantly correlated with both hand and arm kinematics (percentages for monkeys C and G shown separately). Human experiments have also achieved high dimensional control using only one or two 96-channel Utah arrays (Wodlinger et al., 2015). This is efficient for BMI, given that neuron counts are a limiting factor in decoding. It is also significant to our understanding of movement coding: these data suggest M1 utilizes a distributed, higher-dimensional control scheme, and that most M1 units are not tied to just one muscle or kinematic parameter.

The question of whether M1 encodes “intrinsic” (muscle activity) or “extrinsic” (movement direction, limb position in space) variables has been long standing. Though decoders based on extrinsic variables have demonstrated impressive performance, they lack a mechanistic explanation. On the other hand, intrinsic models have recently been accumulating evidence. A dynamic network model incorporating limb and muscle biomechanics (Lillicrap and Scott, 2013) produced distributions of preferred directions that matched those obtained experimentally from monkey reaches in that and other studies (Mitsuda and Onorati, 2002; Suminski et al., 2015). This included a bimodal distribution similar to muscle preferences for movements toward and away from the body. Data from the latter study is shown in Figure 1B. Here, monkeys performed an instructed-delay, center-out reaching task, while signals were recorded with electrode arrays in M1 and premotor areas. The experimenters found non-uniform distributions of preferred directions that consistently correlated with intrinsic differences in muscle activity and arm joint forces at those particular points in the reaches. These shifts in preference would not be expected if the neurons were tuned to extrinsic reach direction, lending evidence to the “intrinsic” hypothesis.

Additionally, it is possible to predict myoelectric (EMG) signals in the arm using the activity of certain M1 units (Pohlmeyer et al., 2007; Ethier et al., 2012; Oby et al., 2013). Zhuang et al. computed joint cross-correlations between neurons and surface EMG of arm muscles in monkeys performing center-out reaching or touchpad pressing (Zhuang et al., 2014). They showed unit-EMG cross-correlations were time-varying, involved multiple significant muscle interactions per unit, and did not always have opposite signs for antagonistic muscles, further indicating the correspondence between M1 neurons and muscles is distributed and dynamic. Despite these complexities, it is possible to generate realistic EMG from neural network models (Sussillo et al., 2015; DePasquale et al., 2016). Sussillo et al. trained recurrent neural networks to reproduce EMG signals from monkeys performing a delayed reach maze task with very low error rates (Figure 1C). They did not train based on actual neural data, or impose any restrictions based on our knowledge of cortical connectivity. Nonetheless, the networks' behavior mimicked neural dynamics at both individual neuron and population levels; model units exhibited varied firing patters that matched those found in actual M1 and PMd neurons, including features of strong preparatory activity, large modulation around move onset, and oscillatory activity around movement. Another study (Overduin et al., 2015) decomposed EMG and neural activity into recurring snippets (“spatiotemporal synergies”), and showed that neural and muscle synergies shared many features, including dimensionality, and timing features.

Overall, approaches that relate M1 firing rates to patterns of muscle activation appear to be a fruitful area for further study, though the dynamics are more complex than a linear neuron to muscle relationship. Even researchers studying corticomotoneuronal cells—the subpopulation of M1 cells that have monosynaptic connections with motoneurons—found that these cells often had preferred directions that differed from their target muscle, and instead appeared to be tuned to a particular function of the muscle (such as agonist or antagonist activation; Griffin et al., 2015).

All of this still leaves the broader question of how the motor system initiates and executes a movement. Renewed interest in the dynamical systems perspective (for review, see Shenoy et al., 2013) has led to some interesting findings. By reducing the dimensionality of a many-neuron data set, it is possible to observe the trajectory of the system through a state space that encompasses preparation and movement epochs. This approach employs the idea that there are many more neurons in M1 than muscles in the system it controls, meaning that a smaller number of meaningful dimensions should be identifiable within the data. Novel dimensionality reduction methods were developed that revealed a rotational structure to reaching trajectories (Churchland et al., 2012), suggesting non-periodic movements like reaches may be controlled in a similar way to rhythmic movements like walking, using a central pattern generator. It has most recently been shown that reach kinematics are well-represented in low-dimensional dynamics of M1 (as well as PMd and PMv; Aghagolzadeh and Truccolo, 2016), and as some had predicted, decoding from these low-dimensional trajectories produced higher performance than decoding from the entire recorded neuronal population. Given the excess of dimensions produced by large neuronal populations, it logically follows that some dimensions will be output-null, and recent work has shown this to be the case (Kaufman et al., 2014), with output-null dimensions allowing for preparatory activity to take place within the same ensembles as movement-generating activity (Figure 1D). This was demonstrated by recording percutaneous EMG and neural activity during a delayed-reach maze task, identifying output-null and output-potent dimensions relative to the EMG activity, and then observing the neural activity in those dimensions. Both dimensions contain strong activity during the movement period, but only the output-null dimension contains activity during preparation. This is expected based on the theory that output-null dimensions allow for preparation without muscle activity. This explanation for the gating of movement onset supplants previous theories involving an inhibitory gating population of cells in M1, and was further supported by single unit recordings (Kaufman et al., 2013).

Finally, it is important to keep in mind the power of adaptation when interpreting findings on neural tuning properties from BMI experiments. Since we know that M1 neurons are capable of modulating their firing properties to fulfill the requirements of the task, it is difficult to say in many cases whether tuning properties are truly native to cells or have been changed by learning, particularly when animals are “overtrained” on tasks to achieve maximum possible decoder performance. In other words, these experiments show how neurons can be tuned, not necessarily how they are always tuned. The aforementioned experiments with simultaneous EMG and cortical recordings are less susceptible to this problem, as the monkeys continue to use the native arm and there is much less need to adapt than when they are using only cortical control. On the other hand, closed-loop brain-controlled BMI is a great opportunity for researchers interested in studying adaptation and motor learning, as discussed in the next section.

### Motor learning

Sensorimotor learning involves learning new mappings between motor and sensory variables (Wolpert et al., 2011), whether those mappings represent the interactions between your fingers and some piano keys, or motor signals mapped directly from cortex with a BMI. As mentioned in previous reviews (Hatsopoulos and Donoghue, 2009; Orsborn and Carmena, 2013), BMI experiments create a direct, causal link between recorded cortical activity, and behavior via the decoding algorithm, allowing for a relatively closed system investigation of motor learning and plasticity. Recently, investigators have been interested in local M1 network changes in response to learning.

**Figure 2:**
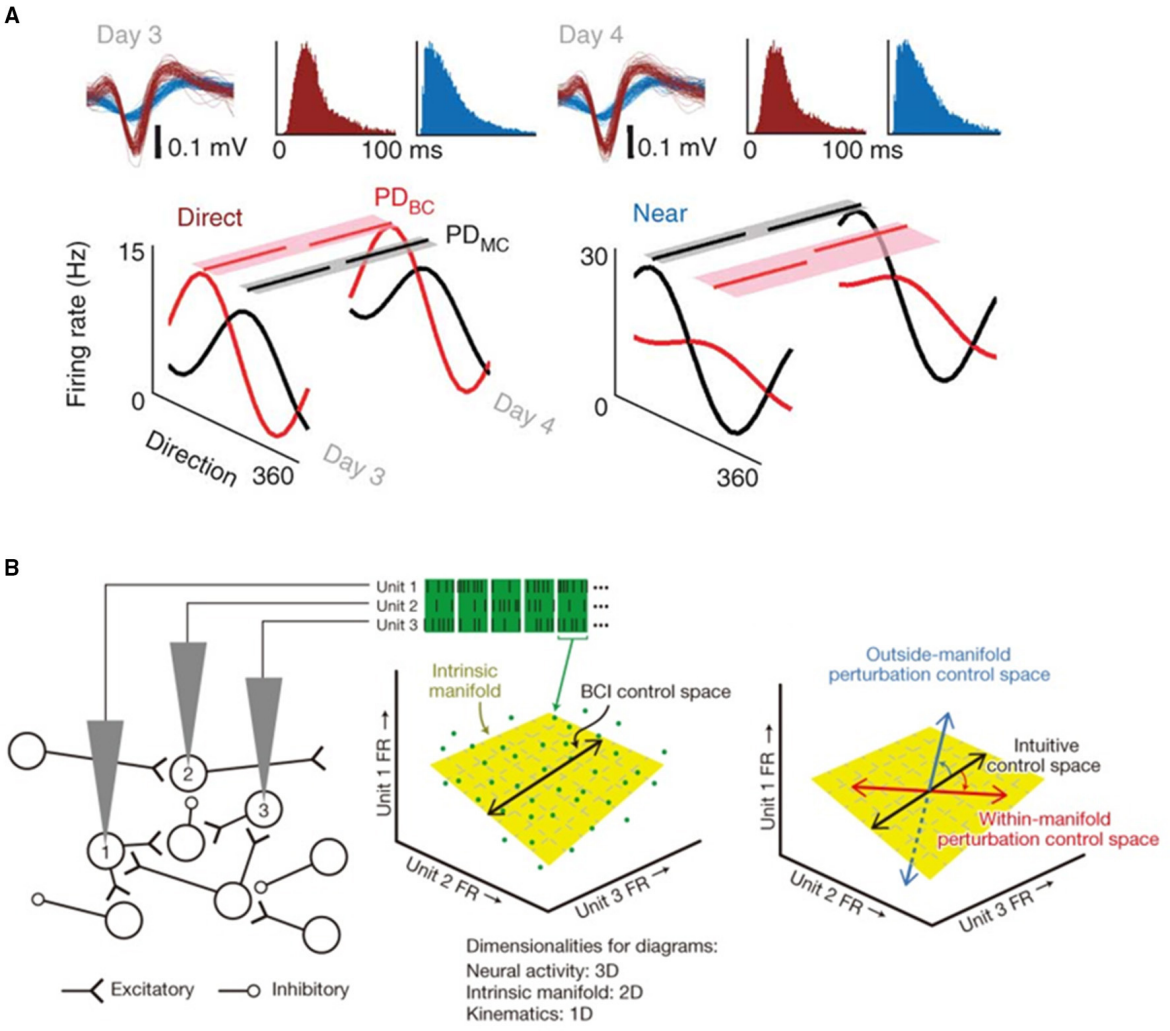
**(A)** Average directional modulation relationship for a direct (mapped) and near (close by but unmapped) unit during manual control and brain control on 2 consecutive days. Partial lines above each tuning curve represent the respective preferred direction for each daily brain control (PD_BC_) and manual control (PD_MC_) session. The shaded region is the respective variance of the bootstrap distributions of PD_BC_ and PD_MC_. Waveforms and interspike interval distributions from a direct (red) and near (blue) unit on consecutive days are also shown. Reprinted with permission of Macmillan Publishers Ltd., from Ganguly et al. (2011), copyright 2011. **(B)** Within-manifold perturbations can be quickly adapted to. The firing rate (FR) observed on each electrode in a brief epoch define a point (green dots) in the neural space. The intrinsic manifold (yellow plane) characterizes the prominent patterns of co-modulation. Neural activity maps onto the control space (black line) to specify cursor velocity. Right, control spaces for an intuitive mapping (black arrow), within-manifold perturbation (red arrow), and outside-manifold perturbation (blue arrow). Adapted with permission of Macmillan Publishers Ltd., from Sadtler et al. (2014), copyright 2014.

Error-based learning (also called adaptation) is used to correct motor behaviors that have gone off track by some perturbation or change in environment. If the neural system recognizes a directional error in, for example, a reach to target, it can attempt to quantify the gradient of the error and adjust the subsequent trajectory to compensate. While this method is fast and can reduce average reach error, there is no well-known mechanism to further improve performance, for example by reducing the variance of the trajectories. It is possible to envision changes in population vectors after changing units' directional contributions (Jarosiewicz et al., 2008; Chase et al., 2012) in this light, as this experiment mimics a visuomotor transformation where error-based learning would be employed. These studies have shown that the vast majority of the correction at the single unit level comes from re-aiming—that is, if the perturbation moves the target 45°, the system will aim at –40° to end nearer the target. Some re-tuning (changes in the directional tuning of units) was also seen, but it accounted for a much smaller portion of the overall correction in this experimental paradigm, which was completed on a short time scale (one experiment of several hundred trials). Over a longer time scale, changes in individual unit tunings become more significant. Ganguly et al. (Ganguly and Carmena, 2009; Ganguly et al., 2011) demonstrated the creation of new cortical maps following perturbation—tuning curves developed, deepened and then remained well-tuned for more than a week (Figure 2A). They also showed that neurons not directly used in decoders underwent changes in tuning depth, though they were smaller than in directly used cells, and that multiple maps could be stored by the same population of cells, indicating widespread changes in motor cortical activity.

Utilizing the idea that neuronal firing rates can be transformed into a low-dimensional subspace that captures important activity patterns, Sadtler et al. found that monkeys could more easily adapt to perturbations within the original neural firing rate space, or “manifold” (Sadtler et al., 2014, Figure 2B). Perturbations outside the established manifold could not be overcome within the time course of one experiment, indicating that within-manifold learning resembles rapid adaptation, while outside-manifold learning may require more involved long-term processes. When a motor problem becomes more complex than a trajectory adjustment, the system might develop an optimal strategy by employing a process such as reinforcement learning (RL), which generates predictions about possible strategies and refines them via error feedback. Such processes have the capacity to reduce the variance of movement trajectories, and to attribute sources of error to individual units. It is difficult to make statements about the role of M1 itself in complex motor learning while cutting the cerebellum, basal ganglia, posterior parietal cortex, and other areas known to be involved, out of the loop. Corticostriatal pathways may enable learning of more arbitrary BMI control associations (Koralek et al., 2012). Additionally, work from other groups has shown that M1 neurons can rapidly modulate their activity to more arbitrary requests when addressed individually (Law et al., 2014).

Nonetheless, M1 single units have been found to be modulated by reward expectation, a crucial component of RL, strongly enough to correctly classify reach trials based on reward (Musallam et al., 2004; Marsh et al., 2015). Legenstein et al. tested a potential mechanism for the RL process in M1 by applying a reward-modulated Hebbian learning rule to a two layer network model (Legenstein et al., 2010), and showing that it produced preferred direction shifts comparable to monkey data. An important feature of the learning rule was that (realistically) noisy neuronal output is used to promote exploration of solutions, which is critical to optimize performance in RL. One sequence of experiments is working toward improved prosthetic control by developing an RL actor-critic neural network decoder and showing that it could be used offline and online to control reaching, that it maintained performance over several weeks, and that it could adapt to cope with perturbations to the neural data (Mahmoudi et al., 2013; Pohlmeyer et al., 2014). Further evidence of motor learning processes can be gleaned from an experiment in use-dependent learning, which utilizes a forward model with priors to change the state of motor system solely through repetition, with no error feedback necessary. In this case, the system makes use of a forward model, either because sensory feedback comes too slowly to be of help, or because the motor task is so novel that other approaches can't be used. Verstynen and Sabes developed an adaptive Bayesian model featuring Hebbian learning that mimicked the variance and directional biases of reaches made by humans (Verstynen and Sabes, 2011). Such a Bayesian estimator could also be of use in RL paradigms, where error signals are used to update the prior distribution.

### Somatosensation

A rapidly growing area of BMI research is the development of sensory feedback approaches for upper limb prosthesis users, also called bidirectional BMIs. An action like an arm reach-to-grasp requires the integration of visual, proprioceptive, and tactile information from multiple regions (Sabes, 2011). Lack of sensation other than visual feedback leads to poor control in myoelectric prosthesis users as well as humans using intracortical BMI systems (Wodlinger et al., 2015). There have recently been several exciting demonstrations of somatosensory replacement: peripheral sensory nerves have been stimulated to provide tactile sensations to human amputees using myoelectric prostheses (Tan et al., 2014; Davis et al., 2016; Schiefer et al., 2016), and intracortical microstimulation (ICMS) has been used to provide virtual tactile signals in monkey experiments (O'Doherty et al., 2011, 2012; Berg et al., 2013; Tabot et al., 2013). The work in ICMS of monkey S1 was built upon earlier work from Romo and colleagues (Romo et al., 1998, 2000) showing that the animals could discriminate between ICMS pulse trains of different frequencies.

Some of these studies use a biomimetic approach, taking advantage of somatotopy and our knowledge of sensory coding in S1 to design stimulation to be as natural as possible. This line of work has led to insights about S1 processing of vibrotactile stimuli, an attractive modality of somatosensation to study, given its robust S1 responses and importance for interacting with textured objects or surfaces. We have learned that a subpopulation of S1 neurons multiplex information about high frequency fingertip vibration by simultaneously representing the amplitude of vibration in instantaneous firing rate and the frequency of vibration by precise phase-locking of spikes (Harvey et al., 2013), and that these two information streams come from different sensory afferents (Saal et al., 2015). The other approach to sensory feedback takes advantage of sensorimotor learning and plasticity. In one experiment, monkeys learned to interpret and use multichannel ICMS in S1 representing proprioception to reach to non-visible targets, and could integrate ICMS and visual feedback to achieve better performance on visible targets (Dadarlat et al., 2015). The animals were able to efficiently integrate a novel and unnatural sensory input (ICMS represented a continuously updating vector between cursor and target position) with a natural input (vision), which is incredibly encouraging for future BMI work. While this study cannot tell us exactly which learning processes led to optimal sensorimotor integration, further research may investigate this issue.

Another area that BMI experiments have continued to elucidate is the sensory content within M1 itself. Neuronal populations in M1 are sensitive to many types of sensory inputs, including tactile as well as proprioceptive. This has been shown in non-human primates (for an excellent and relatively recent review, see Hatsopoulos and Suminski, 2011) and to some extent in humans (Shaikhouni et al., 2013). Many cells are tuned to both sensory and motor variables, though the tunings are not always directionally similar. Tactile somatosensory responses are also fairly robust in M1 (Schroeder et al., 2016), and are tuned differently from proprioception. Proprioception-tuned cells were found to be most tuned to the same or opposite direction as an active reach (Suminski et al., 2009). In studies on fast feedback control—corrective muscle responses occurring just 50–100 ms after a perturbation of the limb (Pruszynski et al., 2011, 2014)—M1 neurons have been documented integrating arm joint information into corrective motor commands within 50 ms, reinforcing that M1 has significant and important sensory processing responsibilities.

## Conclusions

BMI researchers have continued to make significant contributions to sensorimotor neuroscience in the past 10 years. They have uncovered specifics on the structure and organization of M1, elucidated more details on the connection between M1 and muscles, and investigated ensemble control of movement planning and execution. They have explored mechanisms for error-based learning (adaptation), reinforcement learning, and use-dependent learning. Finally, they have catalogd extensive sensory responses in M1 and continue to investigate the role of native sensation to M1 function, since it is likely to be important for continued improvement in BMI performance. As BMI control algorithms continue to improve, tasks can be made more complex. We expect that new experimental paradigms, along with improved recording hardware and analysis techniques, will only accelerate BMI and scientific progress in both humans and non-human primates.

## Author contributions

All authors listed, have made substantial, direct and intellectual contribution to the work, and approved it for publication.

### Conflict of interest statement

The authors declare that the research was conducted in the absence of any commercial or financial relationships that could be construed as a potential conflict of interest.
